# Two-Dimensional Brain Microtubule Structures Behave as Memristive Devices

**DOI:** 10.1038/s41598-019-48677-1

**Published:** 2019-08-27

**Authors:** María del Rocío Cantero, Paula L. Perez, Noelia Scarinci, Horacio F. Cantiello

**Affiliations:** Laboratorio de Canales Iónicos, Instituto Multidisciplinario de Salud, Tecnología y Desarrollo (IMSaTeD), UNSE-CONICET, El Zanjón, Santiago del Estero, Argentina

**Keywords:** Biopolymers in vivo, Biomaterials

## Abstract

Microtubules (MTs) are cytoskeletal structures that play a central role in a variety of cell functions including cell division and cargo transfer. MTs are also nonlinear electrical transmission lines that produce and conduct electrical oscillations elicited by changes in either electric field and/or ionic gradients. The oscillatory behavior of MTs requires a voltage-sensitive gating mechanism to enable the electrodiffusional ionic movement through the MT wall. Here we explored the electrical response of non-oscillating rat brain MT sheets to square voltage steps. To ascertain the nature of the possible gating mechanism, the electrical response of non-oscillating rat brain MT sheets (2D arrays of MTs) to square pulses was analyzed under voltage-clamping conditions. A complex voltage-dependent nonlinear charge movement was observed, which represented the summation of two events. The first contribution was a small, saturating, voltage-dependent capacitance with a maximum charge displacement in the range of 4 fC/μm^2^. A second, major contribution was a non-saturating voltage-dependent charge transfer, consistent with the properties of a multistep memristive device. The memristive capabilities of MTs could drive oscillatory behavior, and enable voltage-driven neuromorphic circuits and architectures within neurons.

## Introduction

MTs are important components of the cytoskeleton that play various roles in cell function, including acting as railways for cargo transfer, and the separation of chromosomes during cell division^1–3^. MTs are hollow cylinders whose wall is built of αβ tubulin heterodimeric units stacked head to tail and aligned into protofilaments^4,5^. Protofilaments are usually incorporated in an MT wall where neighboring subunits are slightly shifted from each other, creating a mosaic, whose corners form distinct nanopores thought to behave as channel-like units that support nonlinear ionic movements^6–9^. Although MTs behave as nonlinear electrical transmission lines^10–15^, no connection has yet been made as to the role nanopores play in the electrical behavior of MTs. MT structures, including 2D sheets^7^ and bundles^8^, generate electrical oscillations whose magnitude depend on both the electric field across the MT surface, and the ionic strength and composition of the surrounding medium. Following the working hypothesis drawn from these findings, electrical oscillations would imply a highly synchronized dramatic change in MT conductance, which requires a gating mechanism for the electrodiffusional ionic movement to ensue.

Memristive devices are based on the concept of the memristor, a two-lead fundamental circuit element correlating changes in charge (*q*) with changes in flux (*φ*), such that memristive properties represent a voltage-dependent resistance as predicted by Leon Chua^16^. Memristors have been an object of scientific interest for the last decade after an experimental device developed by a Hewlett-Packard team^17^. Memristor theory has provided a phenomenological explanation of the Hodgkin-Huxley circuit model of axon function^18^. Gale *et al*.^19^ showed experimentally that memristors spike naturally in a manner qualitatively similar to neurons. Memristors would be appropriate components for neuromorphic architectures^17,20^.

To explore the possible mechanistic steps that drive MT structures to electrically oscillate, the present study was conducted on electrically silent (i.e. non-oscillating) rat brain MT sheets that were voltage-clamped under gigaseal conditions. Transient currents were collected at different holding potentials of varying amplitude and polarity. The electrical response of the silent MT sheets indicated the presence of both voltage-dependent highly nonlinear capacitance and also novel memristive properties, consistent with a time- and voltage-dependent resistance.

## Results

### Electrical properties of non-oscillating MT sheets

**Figure 1:**
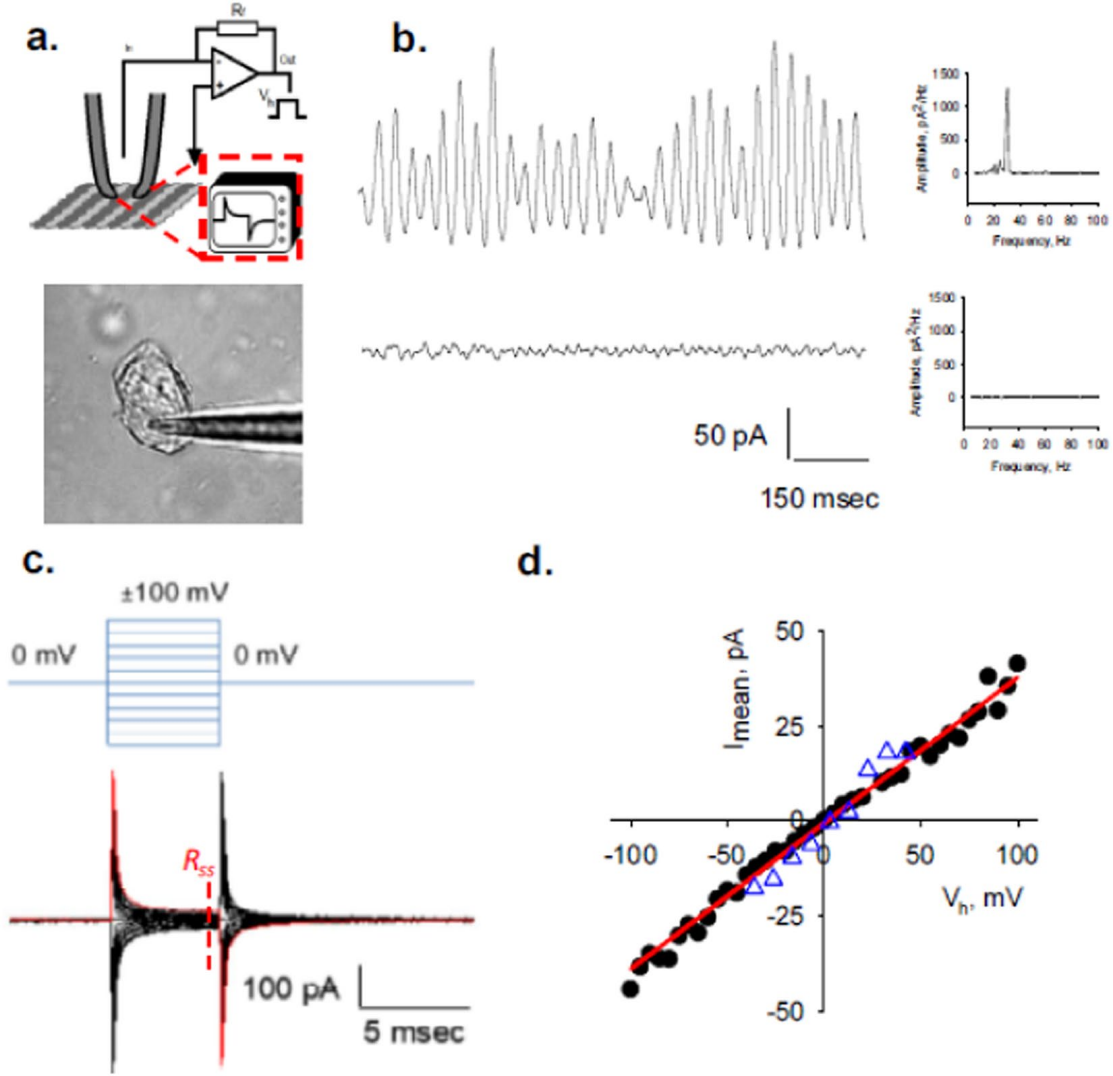
Experimental setup and electrical response of voltage-clamped MT sheet. (**a**) (*Top*) Schematics of electrical setup to obtain electrical signals from giga-sealed MT sheets. (*Bottom*) Rat brain MT sheet with attached patch pipette. (**b**) Top tracing (*Left*), typical oscillations from a patch-clamped MT sheet in symmetrical 140 mM KCl^7^. Bottom tracing is a typical record of a silent MT sheet under similar experimental conditions. Only “silent” (i.e. non-oscillatory) MT sheets were utilized in the present study. (*Right*) Power spectra from both tracings show the fundamental frequency (29 Hz) only in the active MT sheet. (**c**) Representative electrical response to a 5 msec-voltage clamping protocol between ±100 mV, starting from a holding potential of zero mV (as shown in *Top*). Resting time in between voltage steps was 45 msec. (**d**) MT patch conductance obtained by the current-to-voltage relationship for the tracings shown in (**c**). Values (filled circles) were obtained at the time point indicated by *R*_*ss*_. The solid line indicates the ohmic response as shown from the best fitting to a straight line. Similar values were obtained from 20 msec voltage steps (open triangles). The *R*_*ss*_ for this patch was 2.63 ± 0.04 GΩ.

Electrically “silent” MT sheets were voltage-clamped in symmetrical KCl (140 mM) solution, as previously reported to characterize MT electrical oscillations^7^ (Fig. 1a,b). After gigaseal (>1 GΩ) formation, current transients were recorded in response to trains of voltage steps (*V*_*h*_) between ±100 mV from a holding potential of 0 mV (*n* = 118, Fig. 1c). Applied square pulses induced a rapid current transient response followed by a slower decline in current flow until reaching a steady state current, *I*_*ss*_. Based on the time-dependence of the current response, the most simplistic approach to explore the current deflections over time was to model the voltage response to a parallel RC circuit (please see Materials & Methods). The intrinsic MT sheet resistance (*R*_*ss*_) was obtained from the *V*_*h*_*/I*_*ss*_ (Fig. 1c) for both 5 and 20 msec voltage steps. The current-to-voltage relationship was linear within the entire voltage range (±100 mV, Fig. 1d) with values between 0.39 and 3.23 GΩ and a mean of 1.31 ± 0.13 GΩ (*n* = 23).

**Figure 2:**
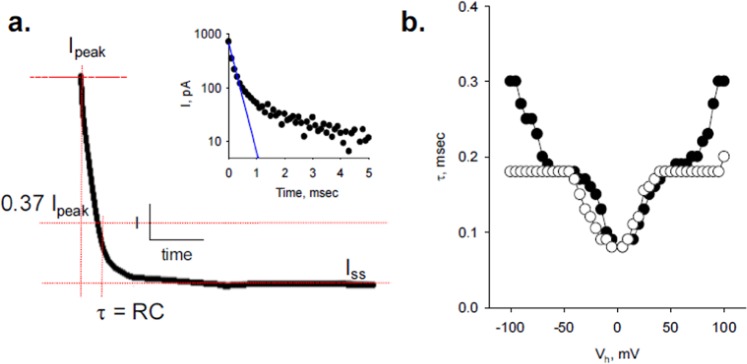
Time-dependence of the relaxation response. (**a**) Current transient obtained in response to the transition from the end of the voltage step to zero mV. Time constants *τ* were obtained at 0.37 *I*_*peak*_ (maximal deflection current) from both the ON and OFF transitions at the onset and ending times of the voltage step, respectively. *Inset*. The relaxation response was multi-exponential, as indicated in the Linear-Log plot. Blue line indicates the mono-exponential function fitting. (**b**) Plot showing voltage dependence of *τ* vs. *V*_*h*_ for the onset (ON, filled circles), and ending (OFF, open circles) of the pulse.

The charge translocated between the voltage step (*t* = 0) at the onset of and the time to reach *I*_*ss*_ represented the total charge during the pulse, which was originally considered to be the capacitive response of the MT sheet at a given *V*_*h*_. Following the original *RC* model, it was hypothesized that at the onset of *V*_*h*_, a large initial current transient would develop that rapidly decreased as the MT sheet capacitance charged. This intrinsic capacitance (*C*_*MT*_) was first explored by calculation of the time constants *τ* for the *V*_*h*_ series (Fig. 2a), which were obtained as the time required for *I*_*peak*_ to reach 37% of its value in the relaxation response. However, time decay of the current tracing did not follow a mono-exponential function (Fig. 2a, Inset), such that this approximation to calculate the translocated charge had limitations, which were emphasized in the *τ* vs. *V*_*h*_ plot (Fig. 2b), showing a complex voltage-dependence for both, the ON and OFF transients at the beginning and end of the pulse, respectively.

**Figure 3:**
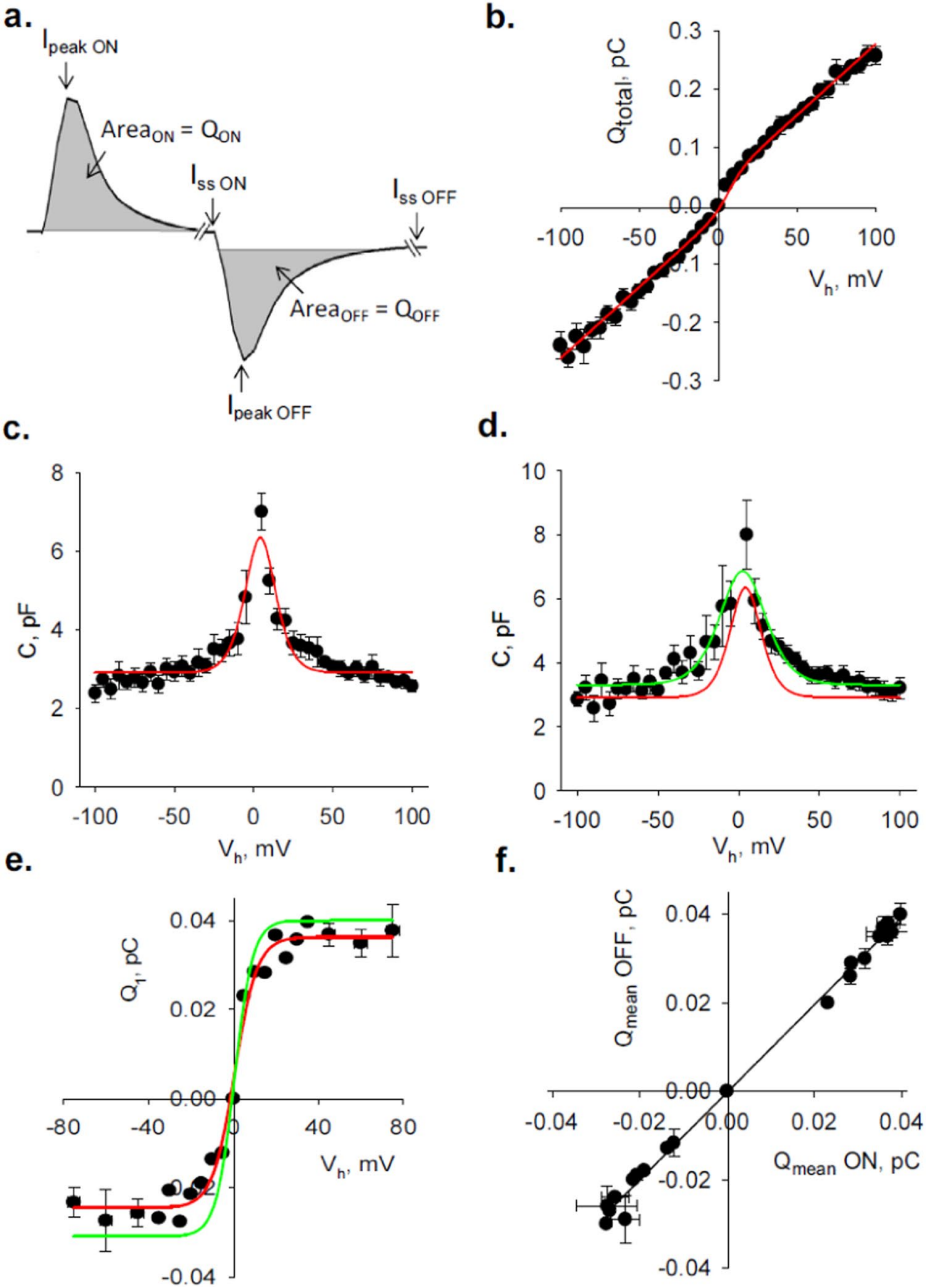
Calculation of displaced charge during current transients. (**a**) Blown up current transients obtained in response to a voltage step. Peak (*I*_*peak*_) and steady state (*I*_*ss*_) current values are shown for both the onset (ON), and ending (OFF) values. Total charge transferred, *Q*_*total*_, (shaded area) for each region was obtained by numerical integration of the total area under the curve. Similar calculations were carried out for each current transient at the onset and ending of the pulse. (**b**) *Q*_*total*_ obtained from experiments conducted in symmetrical KCl (140 mM), was plotted vs. *V*_*h*_, and shown for the entire ±100 mV voltage range. Experimental points (filled circles) are the mean ± SE, of *n* = 9 experiments. The solid line depicts best fitting to Eq. 7. *Q*_*total*_ showed no saturation and a nonlinear voltage response with inflection around zero mV. (**c**) Plot of the patch capacitance *C*_*MT*_ (Eq. 8), as a function of *V*_*h*_. The experimental data (filled circles, *n* = 9), were fitted to Eq. 9 (Red Line). (**d**) Plot of the patch capacitance *C*_*MT*_ (Eq. 8), as a function of *V*_*h*_ after addition of 200 mM K-gluconate to the bathing solution following conditions as before. The experimental data (filled circles, *n* = 6), were fitted to Eq. 9. Solid lines represent best fittings for symmetrical KCl (Red) and asymmetrical (Green) conditions. (**e**) Data in (**b**) were corrected by subtraction of linear response showing the displacement charge vs. *V*_*h*_ and fitted to a Boltzmann function (Eq. 7 without the linear term), as shown by the Red Line under symmetrical conditions and Green Line after addition of K-gluconate. (**f**) The slope of the *Q*_*OFF*_ *vs. Q*_*ON*_ plot indicated the capacitive nature of the translocated charge.

A second method to calculate *C*_*MT*_ was to consider the “area under the curve”, namely the numerical integration of the current decline after *I*_*ss*_ subtraction from each tracing (Fig. 3a). This approach rendered a charge value, *Q*_*total*_, which was independent of the shape of the decaying current. The results for the *V*_*h*_ train evidenced a largely non saturating *Q*_*total*_ vs. *V*_*h*_ curve (Fig. 3b) that was well fitted by Eq. 7, containing both voltage-dependent and independent terms. *C*_*MT*_ was calculated by Eq. 8. The plot of *C*_*MT*_ vs. *V*_*h*_ in symmetrical KCl (140 mM) showed a bell-shape non-linear curve that was fitted by Eq. 9 (Fig. 3c). However, this fitting failed to reproduce precisely the shape of the voltage dependent *C*_*MT*_ between ±25–100 mV. There was slight asymmetry in these regions being lower for negative potentials. To further explore this capacitive distribution, the change in *Q*_*total*_ and *C*_*MT*_ was also explored after further addition of 200 mM K^+^-gluconate to the bath solution. There was a slight change in *Q*_*total*_ that was not clearly distinguishable from the control under symmetrical condition (data not shown). The change in the voltage-dependent capacitance showed a −1.56 mV shift in peak potential (Fig. 3d), which was not statistically significant respect to the control condition (symmetrical KCl, Fig. 3c). However, the response to the increased external ionic strength evidenced wider data dispersion, most clearly at negative potentials, which was consistent with a larger capacitance (Fig. 3d). To further explore the ionic contributions to the voltage-dependent charge displacements, *Q*_*total*_ was corrected by subtraction of the linear response in the *Q*_*total*_ *vs. V*_*h*_ plots (Fig. 3e), unmasking a displacement charge (*Q*_1_) that followed a saturating Boltzmann function (first term of Eq. 7). The saturating charge *Q*_1_ under symmetrical conditions (140 mM KCl) showed values of 0.0363 pC and −0.0245 pC for the positive and negative potentials, respectively, which further supported the asymmetric voltage-dependent charge distribution. However, this symmetry was further shifted after addition of K-gluconate, being the *ΔQ*_*max*_ (difference in fitted values from Fig. 3e, Red and Green lines) in the range of 0.0037 pC and 0.0064 pC for positive and negative potentials, respectively. The results suggested that an excess of impermeable anions, could possibly affect the gating mechanism elicited by capacitive charges. It is important to note that despite ionic asymmetries there was a strict correspondence between *Q*_*ON*_ and *Q*_*OFF*_ (*slope* 0.992 ± 0.016, *r*^2^ = 0.9954, *n* = 12, Fig. 3f) as expected from the capacitive nature of the displaced charge. Nonetheless, the *Q*_*total*_ vs. *V*_*h*_ plot indicated that only a small fraction of charge would respond with a strictly capacitive behavior, a phenomenon inferred by the failure of *τ* = *R*_*ss*_*C*_*MT*_ to show voltage independence (Fig. 2b). This was further supported by the fact that *I*_*ss*_ rendered similar *R*_*ss*_ values for both the 5 msec and 20 msec voltage steps (Fig. 1d). Thus, the complete relaxation response was almost entirely ohmic, indicating the presence of a voltage-dependent dissipative term, consistent with a voltage-dependent resistance that would explain the non-saturating lineal *Q* transferred.

### Memristive properties of the MT sheet

**Figure 4:**
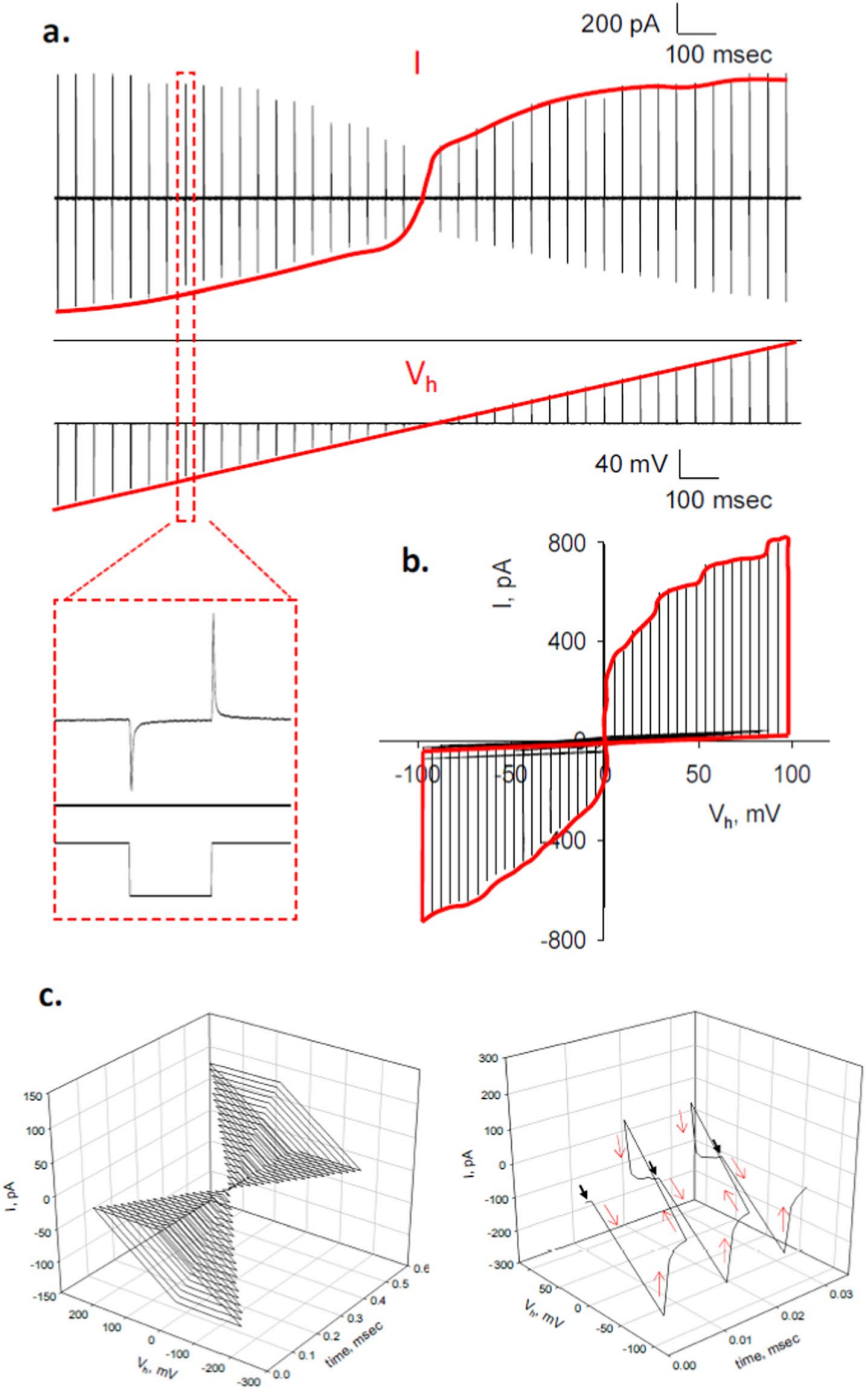
Current-to-voltage relationship of non-oscillating MT sheets. (**a**) Temporal correlation between train of voltage steps between ±100 mV (*V*_*h*_, *Bottom*), and elicited current spikes (*I*, *Top*). Blown up region of a single pair is shown below. (**b**) The plot shows the current-to-voltage response from data in (**a**). Note that each vertical line is actually a spike as shown in the Inset. The solid red line connects the peak values of the spikes, emphasizing the nonlinear, saturating response of *I*_*peak*_ vs. *V*_*h*_. Please note that the portion of linear solid line closed to the abscissa represents the linear, ohmic response of *I*_*ss*_ vs. *V*_*h*_. Data representative of *n* = 9 experiments. (**c**) (*Left*) 3D-plot of an electrical response from a non oscillating MT sheet after ±100 mV train of pulses both holding potential and current response were plotted vs time. (*Right*) The expanded tracing shows the electrical response to −100 mV and −95 mV to indicate the zero current-zero voltage instances as indicated by the black arrows. Red arrows help in following the time response.

The current in response to different voltage steps was next further explored. Current transients were recorded at a sampling rate of 33 kHz to improve the current response of 5 msec square voltage steps. Trains of 5 msec-voltage steps between ±100 mV and 45 msec intervals in between were applied to the MT sheet (Fig. 4a). The linear time series for *V*_*h*_ stimuli (labeled *V*_*h*_ in the Fig.) elicited a temporal, non-linear, saturating current response for *I*_*peak*_, but not *I*_*ss*_ (labeled *I* in the Fig.). This phenomenon indicated the presence of a transient voltage-dependent change in conductance that decayed to a linear response at the end of each respective voltage step (Fig. 4a, Inset). The current-to-voltage plot (Fig. 4b) further showed both, a nonlinear, saturating current response at higher potentials and a pinched profile, namely *I* = 0 pA at *V*_*h*_ = 0 mV. Both observations are consistent with properties expected from memristive devices^21,22^. It should be noted that the vertical lines in Fig. 4b actually are spikes having a peak and a plateau, both emphasized by a red line, which should not be considered a hysteresis cycle. However, the connecting solid red line represents the actual voltage-dependent conductance. Because no cyclical driving function (only square pulses) was applied, we show two 3D plots containing the time, current and voltage parameters (Fig. 4c), where the zero current-zero voltage instances can be observed, as expected.

**Figure 5:**
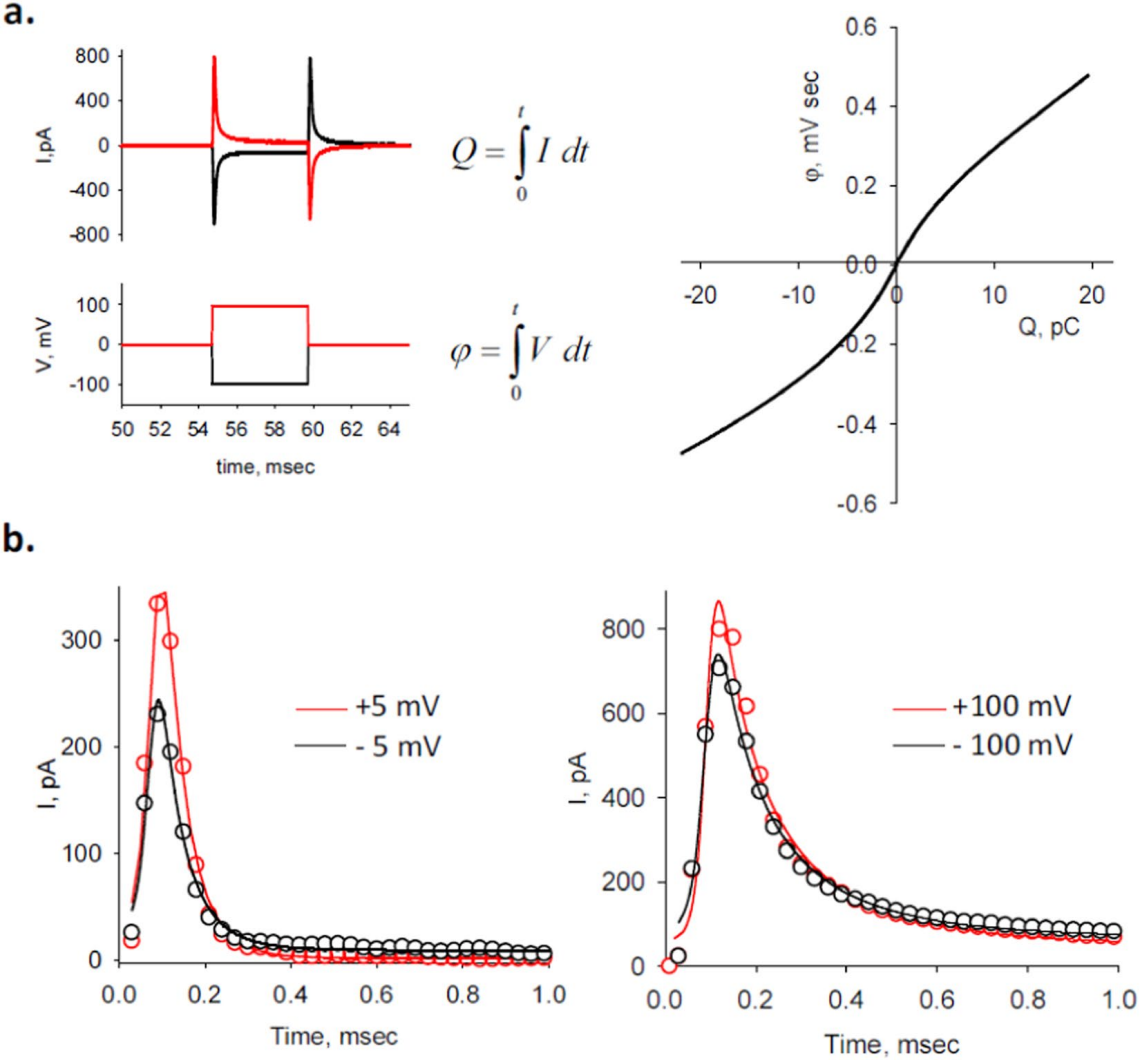
Memristive properties of MT sheet current spikes. (**a**) (*Left*) Schematics of theoretical total charge *Q* (*Top*) and total *φ* (*Bottom*) obtained by integration of either current spikes (Top tracing) or voltage steps (Bottom tracing), respectively. Please note positive pulses are in red and negative pulses in black. (*Right*) Graph showing representative *φ* vs. *Q* plot for voltage steps between ±100 mV. (**b**) Temporal response of current transients for voltage steps between ±5 mV (*Left*) and ±100 mV (*Right*), and best fitted theoretical lines (black and red solid lines, respectively) following Eq. 1. Note that negative currents are shown as positive deflections.

The memristive properties of the MT sheet were further evaluated by calculating and plotting the flux (*φ* = ∫*Vdt*) (Fig. 5a, Left) against the voltage-dependent charge (*Q* = ∫*idt*, without subtraction of ohmic response) (Fig. 5a, Right) that showed at least two defined slopes (*Q* < ±5 pC, and *Q* > ±5 pC), suggesting a behavior similar to a charge controlled memristor^16,23^, capable of producing the spiking response after voltage steps (Fig. 5a)^24^.

To phenomenologically characterize the spiking events after voltage steps, current transients for ±5 mV and ±100 mV (Fig. 5b) were expanded, and found to be well fitted with the equation:

1 $$I(t)={V}_{h}/{R}_{ss}+({V}_{h}/B)t+{V}_{h}/[gt\,\exp (Dt)+ht\,\exp (-\,Jt)]$$

that shares strong similarities with the Mem-Con model of memristive devices that explains neuron spikes^19^. The time and voltage dependent current, *I*(*t*), is defined by the three terms in Eq. 1. The first term represents the constant current at the end of the spike (ohmic), the second term includes a parameter (*B*) that can be thought as to include an inductive component that responds to current changes, and the third term defines the properties of the memristive system^19^, consisting of the sum of two terms representing the memory function, *M*_*e*_, and the conservation function, *R*_*c*_, respectively, where:

2 $$\begin{array}{rcl}{R}_{c}(t) & = & t\,{\rm{\exp }}(Dt)\\ {M}_{e}(t) & = & t\,{\rm{\exp }}(\,-\,Jt)\end{array}$$

and *g, h, D* and *J* are fitting parameters.

## Discussion

MTs are nonlinear electrical transmission lines^10–12^ that produce and conduct electrical oscillations^7,8^. The mechanistic aspects of the MT oscillatory behavior remained heretofore unknown. We originally hypothesized^7^ that the electrical oscillations would require a gating mechanism to enable the highly synchronized electrodiffusional ionic movement through the MT wall. In a sense, the gating mechanism would share similarities with piezoelectric materials such as the piezo-protein prestin, the membrane motor of outer hair cells^25^. Piezo proteins elicit electromechanical transduction^26,27^ such that their mechanical and electrical properties differ upon changes in conformational state^28,29^. This is usually speculated to be a two-state Boltzmann function that shares similarities with the charge displacement observed during the gating process of ion channels^30,31^. The process may be unmasked by the presence of a voltage-dependent capacitance^25–27^. The data herein, however, showed that the electrical response of non-oscillating rat brain MT sheets had a complex voltage-dependent nonlinear charge movement, which represented the summation of two events. The MT surface responded to voltage steps with a nonlinear function containing both voltage-dependent capacitive and resistive components.

The first contribution was a small, saturating, voltage-dependent capacitance, with a maximum charge displacement in the range of 4 fC/μm^2^. This saturating capacitive transient may be associated with a gating step that primes the surrounding electrolyte to the MT wall, and relates this charge to topological features of the MT structure. A small change in the local electric field by charge displacement would modify surface parameters that could include area and thickness, and/or the dielectric properties of the MT. The calculated displaced charges per nanopore, however, would probably be too small to elicit the energetic changes that trigger the electrodiffusional pathway. The major contribution to the electrical response, therefore, likely was a non-saturating voltage-dependent charge transfer, consistent with a multistep memristive device. To further understand this novel property of the MTs, a brief account of memristive properties follows.

Memristance is nonlinear resistance, extended from the concept of the memristor, which is the first, and possibly the only, fundamental non-linear circuit element^32^. The memristor (acronym for resistor with memory) was predicted by Leon Chua^16^ as a basic circuit element, where the memristance *M*(*q*), is the relation d*φ*(*t*)/d*q*(*t*), where *φ*(*t*) and *q*(*t*) are the magnetic flux and charge, respectively. The prediction of this new circuit element from symmetry principles was totally unique and revolutionary, and did not depend on any experimental observation^33^. A broad generalization of memristors to an interesting class of nonlinear dynamical systems called memristive systems was further introduced by Chua in 1976^34^, presenting some interesting properties. While possessing memory and exhibiting small-signal inductive or capacitive effects, neither memristors nor memristive systems store either charge or energy (like a capacitor, for example), but they do “remember” their history because of their changing resistance^34^. A particularly descriptive phenomenon of this element is the “pinched hysteresis loop”, where a memristive system driven by some type of (zero mean) cyclic excitation, the *V-I* plot will be a Lissajous curve, which always passes through the origin. Generally speaking, memristive systems are hysteretic in the sense that at very low frequencies, are indistinguishable from nonlinear resistors while at higher frequencies, they reduce to linear resistors. Chua postulated that memristive systems may explain for example the functioning of the thermistor, the Hodgkin-Huxley membrane circuit model, and the discharge tubes^18,34,35^.

Strukov *et al*.^17^ achieved the making of the first physical memristor from TiO_2_ metal oxide sandwiched between metal electrodes. Other materials have also followed^36–38^. The conductive mechanism(s) of titanium oxide memristors has been ever since an important field of research, which is concerned with their theoretical basis of function and applications. The properties of the memristor can thus be described by modeling TiO_2_ memristors, which are thought to work by the changes in a state variable, *w*(*t*), namely voltage or current, to elicit changes in a material property such as its resistivity, by inter-conversion between its stoichiometric (TiO_2_) and doped (TiO_(2−x)_) forms. Thus, the simplest model considers that *w*(*t*) would change between the stoichiometric and doped forms of TiO_2_ under the influence of an applied voltage. Operationally, when one part of the memristor, say the TiO_2_ increases, the other part, for example the TiO_(2−x)_, decreases. Strukov *et al*.^17^ modeled this phenomenon by the dopant drift model (DDM), a one-dimensional system, such that *M*(*t*) = (*w*(*t*)/*D*)*R*_*ON*_ + (1 − *w*(*t*)/*D*)*R*_*OFF*_, where *D* is the entire length of the device, the first term describes the memristance due to the doped form, and the second one, the amount of material in the stoichiometric form. Interestingly, Pickett *et al*.^39^ utilized an electroformed metal-oxide memristive device to produce a nano-scaled tunneling gap where the electrical current transport process was limited primarily by tunneling, represented by the Simmons equation^40^ in series with the ohmic conduction channel. This model, called the tunnel barrier mechanism (TBM), was able to explain the switching mechanism between the resistance states by changing the width of the tunneling gap that rendered ratios of approximately 500. More recently, Adamatzky’s group^19^ developed the memory-conservation (Mem-Con) theory of the memristor that describes memristance as a magnetostatic function of *φ*(*t*) arising from *q*(*t*) by particularly describing the vacancy current (oxygen atoms in the TiO_2_ memristor). This is a spatially three-dimensional theory of memristive devices, where the memory function (*M*_*e*_) was conceptualized for the electron-driven memristance as *M*_*e*_ = *C*_*m*_*M*. In this model, *C*_*m*_ is an experimental parameter that relates the memristive effects experienced by the vacancies from the electrons to the memristive material and different charge carriers^19^. The memory function describes the doped part of the memristor, and the conservation function describes the stoichiometric part as *R*_*con*_ = [(*D* − *w*(*t*))*ρ*_*OFF*_]/*EF*, where the total memristance, *R*(*t*), is described by *R*(*t*) = *M*_*e*_ + *R*_*con*_. The charge translocation of the non-oscillating MT sheet in our study, followed the behavior of a highly dynamic memristive system, described by a phenomenological equation (Eq. 1). The best fitting model of the electrical behavior of the non-oscillating MT sheet required all three memristor models, DDM-TBM-Mem-Con, and depicts a clear time and voltage dependence of the current spikes upon application of voltage steps in a fashion previously shown for memristive devices^19^. This behavior included an *I*–*V* pinched hysteretic loop, a nonlinear *φ* vs. *q* plot, and an extremely large memristive ratio higher than 1300, consistent with a memristive switch that could sustain oscillations and other operative properties.

In conclusion, the present report provides the first direct evidence for the memristive properties of non-oscillating MT sheets that may underlie the gating mechanism that induces synchronized changes in MT conductance^7^. The advantage for a two-terminal device such as the voltage-clamped MT sheet that stores information without a power source is that it may replace the transistor, a hallmark of our present-day microelectronics^22^. Further, when combined in complex circuits, memristive devices such as MTs could perform logic^41^ and non-traditional computing operations^22,42–46^ in a massively parallel fashion, and with a striking resemblance to brain function.

## Materials and Methods

### MT sheet preparation

Rat brain MT sheets were prepared as recently reported^7^. Briefly, brains were homogenized in a blender with PEM buffer containing in mM: 100 PIPES (pH 6.95), 2.0 EGTA, and 1.0 MgSO_4_ supplemented with 2-mercaptoethanol. The sample was further homogenized with a Teflon-in-glass homogenizer, and then centrifuged at 23.400 *g*. The supernatant (cytosolic extract) was decanted, added GTP (1.0 mM), and incubated for 24 h at room temperature. MT sheets were identified under DIC (Olympus IX71) and immunochemistry with an anti-α tubulin antibody (H-300, sc-5546, Santa Cruz Biotech.) and a secondary bovine anti-rabbit IgG-FITC antibody (sc-2365, Santa Cruz Biotech). Samples were kept frozen at −20 °C until further use.

### Electrophysiological data acquisition and analysis

MT sheets were deposited onto an APTES-coated (3-aminopropyl-triethoxysilane, 02154766, MP Biomedicals) glass surface, and patch-clamped (Fig. 1a) as recently reported^7^. Patch pipettes were made from soda lime capillaries (Biocap, Buenos Aires, Argentina) with a tip diameter of 3–4 μm, and a tip resistance of approximately 14 MΩ. Experiments were conducted under symmetrical ionic conditions, in a saline solution containing, in mM: KCl 140, NaCl 5, EGTA 1.0, and HEPES 10, adjusted to pH 7.18 with KOH. Wherever indicated, similar experiments were conducted after addition of 200 mM K^+^-gluconate to the external surface. Electrical signals were recorded with an Axopatch 200B patch clamp amplifier (Molecular Devices, Sunnyvale CA) and voltage clamp protocols started from zero mV holding potential, and included trains of 5–20 msec voltage steps between ±100 mV. Electrical data were sampled at either 10 kHz or 33 kHz, as indicated. No attempt was made to electronically compensate for pipette capacitance. Data were digitized (Digidata 1440A, Molecular Devices), stored in a personal computer and analyzed with pCLAMP 10.0 (Molecular Devices). Sigmaplot 11.0 (Jandel Scientific, Corte Madera, CA) was used for statistical analysis and graphics. The present study was entirely conducted with “silent” MT sheets showing no spontaneous electrical oscillations (Fig. 1b, Bottom tracing), even at higher holding potentials.

### Equivalent circuit of the “silent” MT sheet

The simplest electrical equivalence of the patch from a “non-oscillating (Fig. 1a)” MT sheet considered the electrical response of the voltage-clamped surface area as a parallel *RC* circuit containing both the intrinsic resistance (*R*_*ss*_) and capacitance (*C*_*MT*_) of the MT wall (Fig. 1c). In such case, the time response of the current, *I*(*t*), after a voltage step (*V*_*h*_) from zero mV would consist of an initial transient that relaxed from a peak current (*I*_*peak*_) to a steady-state value (*I*_*ss*_) with an exponential decay time course, such that

3 $$I(t)=({I}_{peak}\,-\,{I}_{ss})\exp (\,-\,t/\tau )+{I}_{ss}$$

where *t* is the time after voltage stimulation, and the time constant *τ* was quantified as the time taken by the maximal current (*I*_*peak*_) to discharge to 37% of its original value. The parameters *I*_*peak*_, *I*_*ss*_, and *τ* were obtained from experimental data (Fig. 2a).

At steady state, the capacitor (*C*_*MT*_) draws zero current, such that *R*_*ss*_ is given by:

4 $${R}_{ss}={V}_{h}/{I}_{ss}$$

The time constant *τ* related to the transient current was initially assumed to be “capacitive”, such that:

5 $$\tau ={R}_{ss}{C}_{MT}$$

The time interval between consecutive voltage steps was set longer than 5*τ* (experimental *τ* in the range of 0.09–0.3 msec, Fig. 2b) to allow complete charging/discharging of *C*_*MT*_. Experiments conducted with 5 and 20 msec voltage steps showed no significant differences and rendered identical *R*_*ss*_ values (Fig. 1d), indicating the complete discharge of the capacitive response.

### Total charge transferred during current transient

The current-to-time curve was not well fitted by a mono-exponential function (Fig. 2a, Inset, Blue line), suggesting that *τ* was not a reliable parameter to estimate transient charge displacement. In order to improve calculations, the total translocated charge (*Q*_*total*_) was also obtained by integration of the area under the curve from the time integral of the transient current (Fig. 3a), such that

6 $${Q}_{total}={\int }_{0}^{\infty }I(t)dt$$

where *Q*_*total*_ would be the charge accumulated through the entire current transient, which should be independent of the decay function between values, starting with the current at *t* = 0 at the onset of the voltage step.

This process, which was repeated for voltage steps between ±100 mV, rendered a *Q*_*total*_ vs. *V*_*h*_ plot that was largely non-saturating (Fig. 3b), and was best-fitted by a function containing three terms

7 $${Q}_{total}={Q}_{1}\{1/\{1+\exp [\,-\,ze({V}_{h}-{V}_{peak})/kT]\}\}+{C}_{2}{V}_{h}+{Q}_{0}$$

where the first term included a voltage-dependent two-state Boltzmann function, with *Q*_1_ saturating charge representing the maximal transfer, *V*_*peak*_ is the “peak” voltage at half-maximal nonlinear charge transfer, *e* is the electron charge, *k* is Boltzmann’s constant, *T* is absolute temperature, and *z* is the valence. The second term also voltage dependent, represented a linear component, with a non saturating charge, and the third voltage-independent term, *Q*_0_ represented the basal charge component before stimulation. The total MT capacitance (*C*_*MT*_) would then be obtained by:

8 $${C}_{MT}={\rm{d}}{Q}_{total}/{\rm{d}}{V}_{h}$$

### Fitting the capacitance of the MT sheet

The capacitance of the MT wall (*C*_*MT*_) was thus fitted by a model containing voltage-dependent and independent terms that could be described by a two-term equation. The first term includes the first derivative of a Boltzmann function that for simplicity assumed two conformational states differing in both charge density and surface area representing the closed and open states of the gating mechanism^27^. Thus, *C*_*MT*_ would be:

9 $${C}_{MT}=\{{Q}_{1}\times ze\,\exp [-ze({V}_{h}-{V}_{p})/kT]/kT{(1+\exp [-ze({V}_{h}-{V}_{p})/kT])}^{2}\}+{C}_{2}$$

### Ethical statement

All experimental protocols were approved by the Ethics Committee from the Facultad de Odontología, Universidad de Buenos Aires (approved protocol number 014/14, UBA resolution 0082153/2013). All methods were carried out in accordance with approved guidelines.
